# GABA Production by Human Intestinal *Bacteroides* spp.: Prevalence, Regulation, and Role in Acid Stress Tolerance

**DOI:** 10.3389/fmicb.2021.656895

**Published:** 2021-04-15

**Authors:** Nize Otaru, Kun Ye, Denisa Mujezinovic, Laura Berchtold, Florentin Constancias, Fabián A. Cornejo, Adam Krzystek, Tomas de Wouters, Christian Braegger, Christophe Lacroix, Benoit Pugin

**Affiliations:** ^1^Laboratory of Food Biotechnology, Department of Health Sciences and Technology, ETH Zürich, Zürich, Switzerland; ^2^Nutrition Research Unit, University Children’s Hospital Zürich, Zürich, Switzerland; ^3^PharmaBiome AG, Zürich, Switzerland; ^4^Max Planck Unit for the Science of Pathogens, Berlin, Germany; ^5^Laboratory of Molecular Microbiology, Faculty of Chemistry and Biology, University of Santiago, Santiago, Chile; ^6^Laboratory of Human Nutrition, Department of Health Sciences and Technology, ETH Zürich, Zürich, Switzerland

**Keywords:** *Bacteroides*, acid stress tolerance, gut microbiota, GABA, glutamate decarboxylase

## Abstract

The high neuroactive potential of metabolites produced by gut microbes has gained traction over the last few years, with metagenomic-based studies suggesting an important role of microbiota-derived γ-aminobutyric acid (GABA) in modulating mental health. Emerging evidence has revealed the presence of the glutamate decarboxylase (GAD)-encoding gene, a key enzyme to produce GABA, in the prominent human intestinal genus *Bacteroides*. Here, we investigated GABA production by *Bacteroides* in culture and metabolic assays combined with comparative genomics and phylogenetics. A total of 961 *Bacteroides* genomes were analyzed *in silico* and 17 metabolically and genetically diverse human intestinal isolates representing 11 species were screened *in vitro*. Using the model organism *Bacteroides thetaiotaomicron* DSM 2079, we determined GABA production kinetics, its impact on milieu pH, and we assessed its role in mitigating acid-induced cellular damage. We showed that the GAD-system consists of at least four highly conserved genes encoding a GAD, a glutaminase, a glutamate/GABA antiporter, and a potassium channel. We demonstrated a high prevalence of the GAD-system among *Bacteroides* with 90% of all *Bacteroides* genomes (96% in human gut isolates only) harboring all genes of the GAD-system and 16 intestinal *Bacteroides* strains producing GABA *in vitro* (ranging from 0.09 to 60.84 mM). We identified glutamate and glutamine as precursors of GABA production, showed that the production is regulated by pH, and that the GAD-system acts as a protective mechanism against acid stress in *Bacteroides*, mitigating cell death and preserving metabolic activity. Our data also indicate that the GAD-system might represent the only amino acid-dependent acid tolerance system in *Bacteroides*. Altogether, our results suggest an important contribution of *Bacteroides* in the regulation of the GABAergic system in the human gut.

## Introduction

The gut microbiota has emerged as a key player in the development and maintenance of human health. Perturbation of its composition or function has been associated with a wide range of disorders (Fan and Pedersen, 2021). With a collection of genes ~150 times larger than the human genome (Qin et al., 2010), the gut microbiota holds tremendous potential for the biosynthesis and transformation of molecules essential for both microbe and host physiology. Besides the well-studied and health-promoting short-chain fatty acids (SCFA; acetate, propionate, and butyrate), other microbial metabolic pathways greatly contribute to human health, such as the synthesis of essential vitamins, the transformation of bile acids, and the production of a multitude of antimicrobial, signaling, or immunomodulatory small molecules (Donia and Fischbach, 2015; Yoshii et al., 2019). Accumulating evidence has suggested that gut microbes can also produce metabolites with high neuroactive potential (neurotransmitters), including norepinephrine, tryptamine, serotonin, dopamine, and γ-aminobutyric acid (GABA; Strandwitz, 2018; Valles-Colomer et al., 2019). In turn, these microbiota-derived neurotransmitters can modulate host homeostasis within the gastrointestinal tract (GIT), but also at distant body sites such as the brain *via* complex neuronal, immunological, and humoral signaling cascades, i.e., the gut-brain axis (Fischbach and Segre, 2016; Cryan et al., 2019). For most of these neurotransmitters, the exact function and benefits for the bacterial cells, the mechanisms regulating their production within the gut ecosystem, or their precise interaction with intestinal and peripheral tissues remain largely unexplored.

Previous studies have shown that GABA, the major inhibitory neurotransmitter in the brain, can be produced by bacteria *via* two distinct pathways. On the one hand, a series of enzymes can convert arginine, ornithine, and agmatine to putrescine and subsequently GABA, an intermediate for the generation of succinate *via* the GABA shunt pathway, thereby representing a strategy to harvest carbon and nitrogen sources under nutrients-limiting conditions (Shaibe et al., 1985). On the other hand, GABA can be produced *via* the glutamate decarboxylase (GAD) system, with its key enzyme a pyridoxal-5'-phosphate-dependent GAD (encoded by *gadA* or *gadB*) converting glutamate to GABA while producing CO_2_ and consuming a proton (Smith et al., 1992). Combined with an amino acid antiporter (*gadC*), this system was described as an acid resistance mechanism in some bacterial taxa (Feehily and Karatzas, 2013). To date, GABA-production has been mainly studied in the model organism *Escherichia coli* (Capitani, 2003), the pathogen *Listeria monocytogenes* (Cotter et al., 2001) as well as in several *Bifidobacterium* spp. (Pokusaeva et al., 2017) and lactic acid bacteria (e.g., *Lactobacillus* spp., *Lactococcus lactis*, and *Streptococcus thermophilus*; Cui et al., 2020), primarily for the development of probiotics and GABA-containing fermented food with health benefits (Diez-Gutiérrez et al., 2020). Interestingly, recent metagenomic-based studies in mice (Zheng et al., 2016) and in human cohorts (Valles-Colomer et al., 2019) have unveiled a strong correlation between depression and glutamate/GABA metabolism by the gut microbiota, thus instigating further characterization of GABA-producers beyond probiotic strains, including strict anaerobic gut commensals.

A first screening of the Integrated Microbial Genomes/Human Microbiome Project database identified 26 unique bacterial genera harboring *gadB* orthologs, including *Bacteroides*, one of the most abundant and prevalent genera of the GIT (Pokusaeva et al., 2017). This finding was confirmed by Strandwitz et al. (2019) using a set of 1,159 taxonomically diverse gut bacterial genomes, who identified 45 *Bacteroides* strains harboring *gadB* orthologs, with GABA production confirmed for six strains. Despite this genetic evidence and initial phenotypic data, a comprehensive molecular and physiological analysis of the prevalence and species/strain variability of GABA production in *Bacteroides*, as well as the benefits of GABA production to the cells are still lacking; all of which are critical aspects to understand the contribution of microbiota-derived GABA in modulating host homeostasis.

Here, we report the production of GABA in a range of *Bacteroides* strains from human intestinal origin (representing 11 species) using culture and metabolic assays combined with comparative genomics and phylogenetics. We show that GABA production is a common trait in human *Bacteroides* strains (although produced levels are strain-specific) owing to a highly conserved GAD-system composed of a GAD, a glutaminase, a glutamate/GABA antiporter, and a potassium channel. We identified glutamate and glutamine as primary and secondary precursors, respectively, and demonstrate that GABA production is regulated by pH, and acts as a protective mechanism against acid stress in *Bacteroides*.

## Materials and Methods

### Bacterial Strains

A panel of 17 *Bacteroides* strains isolated from human intestine including several strains of the same species was acquired from the German Collection of Microorganisms and Cell Culture GmbH (DSMZ, Braunschweig, Germany) and the strain collection of PharmaBiome AG (Zürich, Switzerland; Table 1). Two additional strictly anaerobic putative GABA producers from the human gut, i.e., *Eubacterium limosum* BT-4119 (own collection) and *Parabacteroides distasonis* PB-SUZFK (PharmaBiome AG; Table 1) were also included in the *in vitro* screening. Reference strains thereof were subsequently used to root the 16S rRNA, GAD-system genes, and genome phylogenetic trees. Strains were stored at −80°C in 25% (v/v) anaerobic glycerol stocks, and routinely cultivated in Hungate tubes containing anaerobic medium (described below) at 37°C without shaking. The procedures for anaerobic medium preparation in Hungate tubes was performed as previously described (Bircher et al., 2018).

**Table 1:** Bacterial strains screened for GABA production.

Genus	Species	Strain	Culture collection	Isolation source	Genbank accession number (16S rRNA gene)
*Bacteroides*	*caccae*	DSM 19024	DSMZ	Human feces	AB510697
*Bacteroides*	*dorei*	PB-SNPAX	PB	Human feces	MT749285
*Bacteroides*	*faecis*	DSM 24798	DSMZ	Human feces	GQ496624
*Bacteroides*	*faecis*	PB-SESWS	PB	Human feces	MT749282
*Bacteroides*	*fragilis*	DSM 2151	DSMZ	Appendix abscess	NR_074784
*Bacteroides*	*fragilis*	PB-SZSJC	PB	Human feces	MT749278
*Bacteroides*	*intestinalis*	DSM 17393	DSMZ	Human feces	AB214328
*Bacteroides*	*ovatus*	DSM 1896	DSMZ	Human feces	EU136682
*Bacteroides*	*plebeius*	PB-SLKZP	PB	Human feces	MT749279
*Bacteroides*	*thetaiotaomicron*	DSM 2079	DSMZ	Human feces	NR_074277
*Bacteroides*	*uniformis*	DSM 6597	DSMZ	Human feces	AB050110
*Bacteroides*	*uniformis*	PB-SWTWH	PB	Human feces	MT749281
*Bacteroides*	*uniformis*	PB-SARUR	PB	Human feces	MT749280
*Bacteroides*	*uniformis*	PB-SMSXL	PB	Human feces	MT749284
*Bacteroides*	*vulgatus*	DSM 1447	DSMZ	Human feces	AB510712
*Bacteroides*	*vulgatus*	PB-SZEJJ	PB	Human feces	MT749283
*Bacteroides*	*xylanisolvens*	DSM 18836	DSMZ	Human feces	AM230650
*Eubacterium*	*limosum*	BT-4119	FBT	Human feces	MT749287
*Parabacteroides*	*distasonis*	PB-SUZFK	PB	Human feces	MT749286

DSMZ, German Collection of Microorganisms and Cell Culture GmbH; PB, PharmaBiome AG; FBT, Laboratory of Food Biotechnology, ETH Zürich.

### *In vitro* Screening for GABA Production

All strains were reactivated from glycerol stocks by a 1.25% (v/v) inoculation of anaerobic modified yeast extract casitone and fatty acid medium (mYCFA; O_2_-free CO_2_ head gas), consisting of (L^−1^): 10 g amicase (Sigma-Aldrich, St. Louis, MO, United States), 2.5 g yeast extract (Sigma-Aldrich), 4 g NaHCO_3_, 0.45 g K_2_HPO_4_, 0.45 g KH_2_PO_4_, 0.9 g NaCl, 0.9 g (NH_4_)_2_SO_4_, 90 mg MgSO_4_, 90 mg CaCl_2_, 10 mg hemin, 1 g L-cysteine·HCl, 10 μg biotin, 10 μg cobalamin, 30 μg *p*-aminobenzoic acid, 50 μg folic acid, 150 μg pyridoxamine, and 5.75 ml of volatile fatty acids solution (Bircher et al., 2018). Medium was supplemented with glucose (33 mM) and pH was adjusted to 6.5. Pre-cultures were incubated for 24 h. The screening was initiated by inoculating 1.25% (v/v) of pre-culture in 8 ml of fresh mYCFA or mYCFA supplemented with 61 mM glutamate (mYCFA-Glu). After 48 h of incubation at 37°C, optical density (OD_600_) and pH were measured, and 1 ml of bacterial culture was centrifuged (14,000 × *g*, 10 min, 4°C). Supernatants were directly processed for quantifying GABA and other molecules (amino acids, biogenic amines, glucose, SCFAs, and intermediate metabolites) as described below. Each condition was screened using three independent replicates. The GABA overall yield coefficient (Y_GABA/Glu_; mol/mol) was calculated after 48 h incubation as the amount of GABA produced over glutamate consumed in mYCFA or mYCFA-Glu.

### GABA Precursors Utilization and Production Kinetics

*Bacteroides thetaiotaomicron* DSM 2079 was cultured in minimal medium (MM) to investigate growth and GABA production kinetics in the presence of primary (glutamate) and secondary (glutamine, α-ketoglutarate) GABA precursors, and to prevent possible interferences with complex components in mYCFA. Anaerobic MM (O_2_-free N_2_ head gas) was slightly modified from Tramontano et al. (2018) and consisted of (L^−1^): 5 g glucose, 13.6 g KH_2_PO_4_, 0.88 g NaCl, 1.12 g (NH_4_)_2_SO_4_, 0.38 mg FeSO_4_·7H_2_O, 9.52 mg MgCl_2_, 30.2 mg CaCl_2_, 10 mg hemin, 31 mg L-histidine, 0.5 g L-cysteine·HCl, 5 μg cobalamin, and 1 mg menadione. pH was adjusted to 6.5. After 48 h reactivation in mYCFA, pre-cultures of *B. thetaiotaomicron* DSM 2079 were transferred (1.25%, v/v) into MM, grown to an OD_600_ of ~0.6, and re-inoculated (1.25%, v/v) into MM, MM + 10 mM glutamate (MM-Glu), MM + 10 mM glutamine (MM-Gln), or MM + 5 mM α-ketoglutarate to initiate the kinetic analysis. OD_600_, metabolite and substrate concentrations (glutamate, glutamine, glucose, GABA, and SCFA), and pH were monitored during 72 h culture at 37°C. Each condition was assessed using three independent replicates. Maximum specific growth rate (μ_max_) was calculated according to first order growth kinetics, and the overall yields of Y_GABA/Gln_ and Y_GABA/Glu_ (mol/mol) were calculated as the amount of GABA produced over glutamine (MM-Gln) or glutamate (MM-Glu) consumed after 72 h incubation, respectively.

### Acid Stress Tolerance Assay and Cell Viability

The effect of acid stress on GABA production and survival of *B. thetaiotaomicron* DSM 2079 was tested after reactivation of glycerol stock in mYCFA for 48 h, and inoculation (1.25%, v/v) into MM followed by incubation for 15 h to reach late exponential phase (OD_600_ ~1.5). In an anaerobic chamber (10% CO_2_, 5% H_2_, and 85% N_2_, Coy Laboratory Products Inc., Ann Arbor, MI, United States), 1 ml bacterial culture was harvested, centrifuged (14,000 × *g*, 5 min, room temperature), and washed with sterile 0.9% NaCl solution. Acid stress was induced by resuspending cell pellets in 1 ml MM, MM-Glu, or MM-Gln, at four different pH conditions each, i.e., 6.3, 5.5, 4.1, or 3.1, followed by anaerobic incubation at 37°C. The pH, cell viability, and GABA concentration were measured after 1 h of incubation. Each condition was tested in three independent replicates.

Cell viability was determined by membrane integrity analysis using flow cytometry with two different dyes, both staining nucleic acid: the cell-impermeable molecule propidium iodide (PI, Life Technologies, Zug, Switzerland), only entering cells with a damaged membrane, and the cell-permeable SYBR Green I stain (SG, Life Technologies). Viable count *via* flow cytometry was previously shown to correlate closely with agar plate counting (Ou et al., 2017), with viable but nonculturable cells also quantified by the former. Staining and subsequent flow cytometric analysis was performed as previously described (Bircher et al., 2018). Briefly, bacterial cultures were diluted with filtered phosphate buffered saline (PBS) solution to achieve an approximate concentration of 10^7^ cells/ml. Resulting dilutions (30 μl) were then mixed with 267 μl PBS, and 3 μl SG (1x conc.) to assess total cell counts, or with 3 μl SGPI solution (SG: 1x conc.; PI: 4 μM) to assess intact cells. Mixtures were incubated 20 min at room temperature in the dark. Samples were analyzed with a Cytomics FC 500 (Beckman Coulter GmbH, Krefeld, Germany) equipped with an air-cooled argon ion laser emitting 20 mW at 488 nm. Data analysis was performed with Kaluza Analysis 2.1 (Beckman Coulter GmbH). Total (SG-stained samples) or intact cells (SGPI-stained samples) were selected using the green and red detection channels. Viability (%) was calculated as the ratio of intact to total cells.

### Metabolites Quantification

#### Amino Acids, Biogenic Amines, and Neurotransmitters

The concentrations of GABA, glutamate, glutamine, and other amino acids and biogenic amines in bacterial supernatants were determined by ultra-performance liquid chromatography equipped with a diode array detector (UPLC-DAD) and pre-column derivatization with diethyl ethoxymethylenemalonate (DEEMM), modified from Redruello et al. (2013). Analytical-grade standard chemicals for agmatine sulfate salt, L-alanine, ammonium chloride, L-arginine, L-asparagine anhydrous, L-aspartic acid, cadaverine dihydrochloride, GABA, L-glutamic acid monosodium salt hydrate, L-glutamine, glycine, histamine dihydrochloride, L-histidine monohydrochloride monohydrate, L-isoleucine, L-lysine, L-methionine, L-ornithine monohydrochloride, L-phenylalanine, L-proline, putrescine dihydrochloride, L-serine, tryptamine hydrochloride, L-tryptophan, tyramine hydrochloride, L-tyrosine, L-valine (Sigma-Aldrich), L-leucine, and L-threonine (VWR International GmbH, Darmstadt, Germany) were used to generate standard curves. Standard stock solutions were prepared at 10 mM in 0.1 M HCl and were further diluted to generate standard curves. The derivatization was performed by mixing 200 μl standard solution or supernatant sample with 350 μl borate buffer (1 M H_3_BO_3_ adjusted to pH 9 with NaOH), 150 μl methanol, 8 μl internal standard (2 g/L L-2-aminoadipic acid, Sigma-Aldrich), and 7 μl DEEMM (VWR International GmbH). The reaction mix was incubated at room temperature in an ultrasound bath for 45 min, and was subsequently heated at 70°C for 2 h to stop the derivatization. Samples were passed through a 0.45 μm nylon membrane filter and stored at 4°C until UPLC-DAD analysis.

An ACQUITY UPLC H-Class system (Waters Corp., Milford, MA, United States) coupled to a diode array detector at 280 nm was used to detect the derivatized molecules. The separation was performed at 40°C using an ACQUITY BEH C18 column (1.7 μm particle size; 2.1 × 100 mm; Waters Corp.). Samples (1 μl injection) were eluted with a gradient of (A) 25 mM acetate buffer (pH 6.6), (B) 100% methanol, and (C) 100% acetonitrile as follows (flow rate 0.46 ml/min): 0–2 min [A 92–93%, B 2–1.5%, C 6–5.5%]; 2–4.5 min [A 93–85%, B 1.5–4%, C 5.5–11%]; 4.5–6.5 min [A 85%, B 4%, C 11%]; 6.5–8 min [A 85–80%, B 4–6%, C 11–14%], 8–12.5 min [A 80–70%, B 6–2%, C 14–28%]; 12.5–15.5 min [A 70–55%, B 2–3%, C 28–42%]; 15.5–18 min [A 55–45%, B 3–1%, C 42–54%]; 18–20 min [A 45–0%, B 1–20%, C 54–80%]; 20–27 min [A 0%, B 20%, C 80%]; 27–28 min [A 0–90%, B 20–2%, C 80–8%]; and 28–30 min [A 90%, B 2%, C 8%]. Detection limit ranged from 0.03 to 0.05 mM for all analytes. Data were processed using Empower 2 software (Waters Corp.).

#### Glucose, Short-Chain Fatty Acids, and Intermediate Metabolites

Glucose and organic acids (i.e., SCFA and intermediate metabolites) were quantified by high-performance liquid chromatography equipped with a refractive index detector (HPLC-RI) as previously described (Poeker et al., 2018). Briefly, 200 μl bacterial supernatant was passed through a 0.45 μm nylon membrane filter prior to HPLC-RI analysis. The separation was carried out with a LaChrom HPLC-System (Merck-Hitachi, Japan) using a SecurityGuard Cartridges Carbo-H (4 × 3.0 mm; Phenomenex Inc., Torrance, CA, United States) connected to a Rezex ROA-Organic Acid H+ (300 × 7.8 mm; Phenomenex Inc.) column. Samples (40 μl injection) were eluted at 40°C under isocratic conditions (10 mM H_2_SO_4_, flow rate 0.4 ml/min), and analytes were quantified using a refractive index detector L-2490 (Merck Hitachi). Data were processed using EZChrom software (Agilent, Santa Clara, CA, United States).

### *In silico* Analysis of the Prevalence, Synteny, and Phylogeny of GAD-System Genes

Genome sequences and associated metadata from *Bacteroides* spp. and other GABA-producing taxa were acquired from the National Center of Biotechnology Information (NCBI) assembly database. As of September 2020, a total of 967 *Bacteroides* genomes were downloaded and subjected to the GToTree workflow (Lee, 2019). Briefly, HMMER (v3.2.1) was used to search each genome for the target 90 single-copy genes (SCG) specific to *Bacteroidetes*, allowing the quantification of genome completion and redundancy scores. Genomes with an estimated redundancy >13% were filtered out and a total of 961 *Bacteroides* genomes (including 40 fully assembled genomes) were used for further analysis. An alignment based on the 90 SCG sequences was generated using MUSCLE (v3.8) and filtered using TrimAl (v1.4). A maximum-likelihood phylogenetic tree was constructed using FastTree (v2.1) which was then visualized together with the metadata using Anvi’o (v6.2). All genomes were annotated with NCBI’s PGAP (Haft et al., 2018) and Prokka (Seemann, 2014). An annotation-based gene catalog including GAD-system genes and additional genes involved in amino acid-dependent acid tolerance systems, as described by Guan and Liu (2020), was established using the following queries: “glutamate decarboxylase,” “glutaminase” and/or “glutaminase A,” “glutamate/gamma-aminobutyrate antiporter,” “two pore domain potassium channel family protein,” “arginine decarboxylase,” “ornithine decarboxylase,” “lysine decarboxylase,” “tyrosine decarboxylase,” “histidine decarboxylase,” and “arginine deiminase.” Analysis of the GAD-system synteny was conducted using the whole genome alignment plugin in CLC Genomic Workbench (v11.0, QIAGEN, Aarhus, Denmark). Operon and transcript features were retrieved from “Theta-Base,” an open access transcriptome database for *B. thetaiotaomicron* DSM 2079 (Ryan et al., 2020). Phylogenetic analysis of 16S rRNA and GAD-system gene sequences was carried out with Molecular Evolutionary Genetics Analysis (MEGA, v7.0). Following multiple sequences alignment with ClustalW, phylogenetic trees were generated with 1,000 bootstrap replications using the maximum-likelihood method and the Jukes-Cantor correction model.

### Data Analysis, Visualization, and Statistical Analysis

Data were analyzed and visualized using GraphPad Prism (v8.2.0) or R software (v3.6.3). The metabolite-metabolite correlation heatmap was produced using *corrplot* (v0.84) and *rstatix* (v0.4.0) packages; significance level was set to *p* < 0.05. Scatter plots were generated using *ggstatsplot* (v0.3.1) package. The bacterial-metabolite heatmap was generated with *pheatmap* (v1.0.12) package using Pearson correlation to calculate rows and columns distance for hierarchical clustering; each metabolite concentration was scaled to *z-scores*.

Normality and homogeneity of variance of the data sets were verified using the Shapiro-Wilk normality and Brown-Forsythe test, respectively. Statistical analysis was performed by one-way ANOVA, including Tukey’s test to correct for multiple comparison. Significance level was set to *p* < 0.05.

## Results

### Production of GABA by Intestinal *Bacteroides* Strains

**Figure 1:**
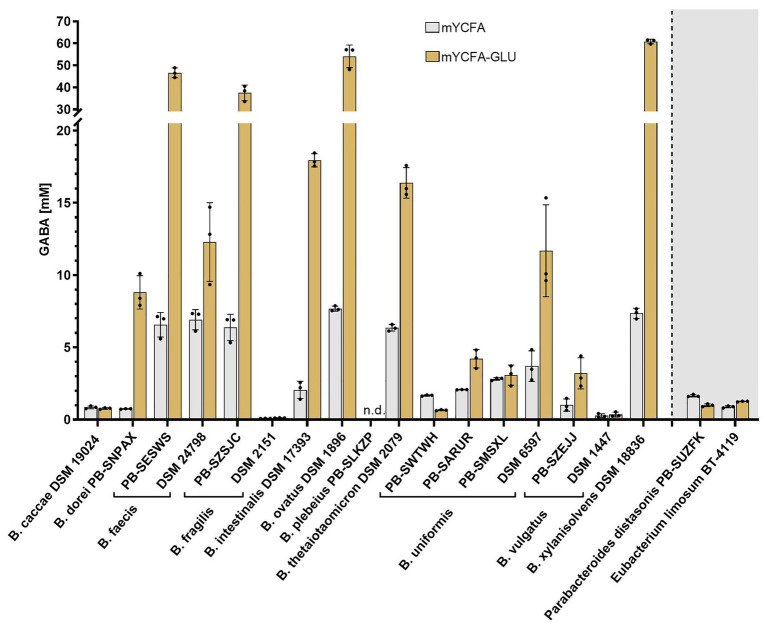
γ-Aminobutyric acid (GABA) production by intestinal *Bacteroides* strains in the presence of two different glutamate concentrations. *Bacteroides* strains were incubated anaerobically at 37°C in mYCFA (8.38 ± 0.27 mM glutamate) and mYCFA-Glu (69.49 ± 1.10 mM glutamate) for 48 h (*n* = 3). GABA quantified in the culture supernatant is displayed. *Parabacteroides* and *Eubacterium* strains were screened identically. Dots represent replicates; bars represent SD. GABA detection limit: 0.03 mM. n.d., not detected.

To assess whether a range of intestinal *Bacteroides* can produce GABA under physiological conditions of the human GIT, a panel of 17 metabolically and genetically diverse human intestinal strains (Supplementary Figure S1) were screened in mYCFA. As previous genetic studies suggested the presence of *gadB* orthologs in *Bacteroides* (Pokusaeva et al., 2017; Strandwitz et al., 2019), the strains were additionally tested in mYCFA supplemented with glutamate (mYCFA-Glu; final glutamate concentrations of 8.38 ± 0.27 and 69.49 ± 1.10 mM in mYCFA and mYCFA-Glu, respectively). All tested *Bacteroides* strains grew well on both media (OD_600_ > 1.1; Supplementary Data File 1), with most strains (16 out of 17) able to produce GABA, generally at higher concentration in mYCFA-Glu compared to mYCFA, except for *Bacteroides plebeius* PB-SLKZP which showed no production under any of the tested conditions (Figure 1). The extracellular concentration of GABA, however, greatly differed among the GABA-producing strains, ranging from 0.09 mM (*Bacteroides fragilis* DSM 2151 in mYCFA) up to 60.84 mM (*Bacteroides xylanisolvens* DSM 18836 in mYCFA-Glu). The highest GABA-producers (>35 mM) were *Bacteroides faecis* PB-SESWS, *B. fragilis* PB-SZSJC, *Bacteroides ovatus* DSM 1896, and *B. xylanisolvens* DSM 18836. The lowest GABA levels (<1 mM) were observed in *Bacteroides caccae* DSM 19024, *B. fragilis* DSM 2151, and *Bacteroides vulgatus* DSM 1447. GABA production was also confirmed in two additional gut commensals, i.e., *P. distasonis* PB-SUZFK and *E. limosum* BT-4119 (Figure 1). Interestingly, for the tested species, the levels of produced GABA were strain-specific within species. This was evident for *B. fragilis*, with strain PB-SZSJC producing 6.38 and 37.48 mM GABA in mYCFA and mYCFA-Glu, respectively, compared to strain DSM 2151 producing only 0.09 and 0.12 mM GABA. Intra-species variations also occurred in *Bacteroides uniformis* (four strains), *B. vulgatus* (two strains), and *B. faecis* (two strains; Figure 1). These variations were independent of growth performance as indicated by OD_600_-normalized GABA values (Supplementary Data File 2).

Next, we evaluated whether GABA production was associated with specific changes of substrate or metabolite concentrations. Since GABA and glutamate are important molecules linking the carbohydrate and amino acid metabolic pathways (Feehily and Karatzas, 2013), the consumption or production of 36 compounds including glucose, SCFA, amino acids, and biogenic amines was measured in the supernatants of mYCFA and mYCFA-Glu cultures (Supplementary Data File 1). As expected, a significant correlation (*r* = −0.99; *p* < 0.001) between glutamate and GABA levels was observed, further highlighting the nature of glutamate as primary precursor of GABA. However, no other metabolites strongly correlated with GABA levels (−0.60 ≤ *r* ≤ 0.65; Supplementary Figure S2). Glutamate was primarily consumed for the production of GABA, with high GABA yield coefficients (Y_GABA/Glu_) ranging from 0.769 to 0.993 (mol/mol) after 48 h for the high producers *B. faecis* PB-SESWS, *B. fragilis* PB-SZSJC, *B. ovatus* DSM 1896, and *B. xylanisolvens* DSM 18836. *B. plebeius* PB-SLKZP was the only strain accumulating glutamate (Supplementary Table S1). The final pH was variable across strains, ranging from 4.8 (*B. vulgatus* DSM 1447, mYCFA) to 6.4 (*B. ovatus* DSM 1896, mYCFA-Glu), and correlated with glutamate (*r* = −0.82; *p* < 0.001) and GABA (*r* = 0.85; *p* < 0.001) levels (Supplementary Figure S3), thus providing initial evidence of the association between GABA production and pH regulation in *Bacteroides*.

### Synteny, Phylogeny, and Prevalence of the *Bacteroides* GAD-System

**Figure 2:**
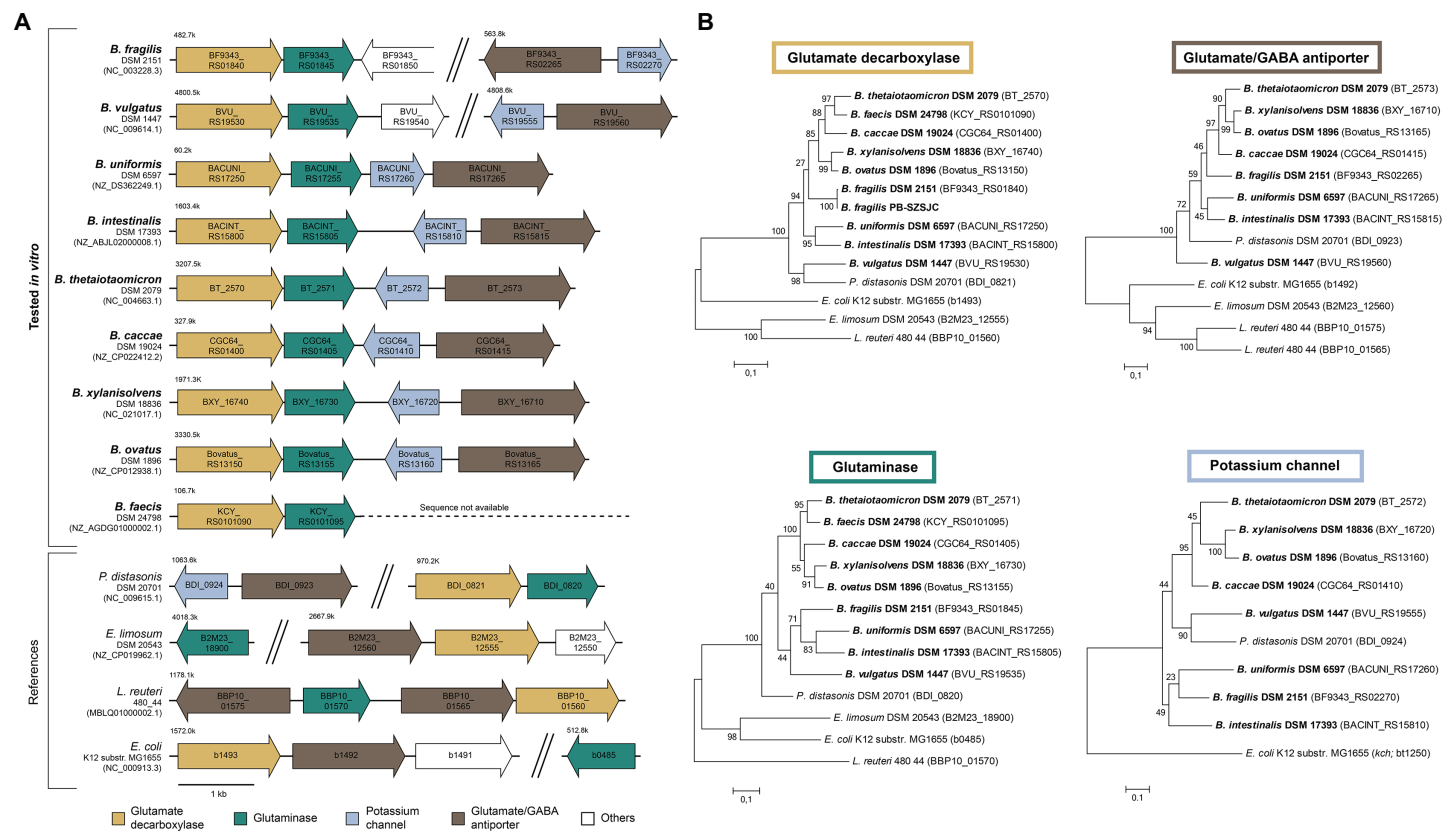
Genetic characteristics of the glutamate decarboxylase (GAD)-system in *Bacteroides* strains. **(A)** Genetic organization of the GAD-system genes (i.e., glutamate decarboxylase, glutaminase, glutamate/GABA antiporter, and potassium channel) in *Bacteroides* strains tested *in vitro* compared to distinct GABA-producing taxa. **(B)** Phylogenetic trees based on GAD-system genes sequences. Trees were generated using the Maximum Likelihood method and the Jukes-Cantor correction model with 1,000 bootstraps. The strains screened *in vitro* are in bold.

We analyzed the *Bacteroides* GAD-system *in silico* to determine whether genetic differences could explain the variation of GABA levels observed *in vitro*. The synteny of the *gadB* region was evaluated in all strains tested *in vitro* whose full genome or scaffold sequences were available. Overall, the organization of the GAD-system is highly similar across the tested *Bacteroides* strains, but substantially different from other GABA-producing taxa like *E. limosum* DSM 20543, *Lactobacillus reuteri* 480_44, and *E. coli* K12 (Figure 2A). In the immediate vicinity downstream of *gadB* (IPR010107 protein family), *Bacteroides* strains harbor a glutaminase-encoding gene (*glsA* ortholog, IPR015868): a unique gene arrangement part of the same operon (confirmed in *B. thetaiotaomicron* DSM 2079 using transcriptome data from “Theta-Base”; Supplementary Figure S4A). The glutamate/GABA antiporter-encoding gene (*gadC* ortholog, IPR022520) is located further downstream and appears to systematically cluster with a potassium (K^+^) channel-encoding gene (IPR028325) in all *Bacteroides* strains, suggesting a functional relationship between these genes (Figure 2A). Additional examination of all *Bacteroides* strains with complete circular genomes (*n* = 40) further confirmed the unique and highly conserved genetic arrangement of the *Bacteroides* GAD-system (Supplementary Figure S5). The phylogenetic analysis of *gadB*, *gslA*, *gadC*, and the K^+^ channel-encoding gene also revealed a high degree of conservation among *Bacteroides* strains at the sequence level, clustering apart from other GABA-producing taxa (Figure 2B). The only prominent variation detected between *Bacteroides* strains lies in the genomic distance between the *gadB*/*gslA* operon and the *gadC*/K^+^ channel-encoding gene cluster, potentially affecting gene co-regulation due to proximity (Korbel et al., 2004). However, a large separation between these regions occurs in the two low GABA-producers *B. vulgatus* DSM 1447 and *B. fragilis* DSM 2151, but not in *B. caccae* DSM 19024, thereby providing insufficient genetic evidences to explain the discrepancy of GABA produced *in vitro*.

**Figure 3:**
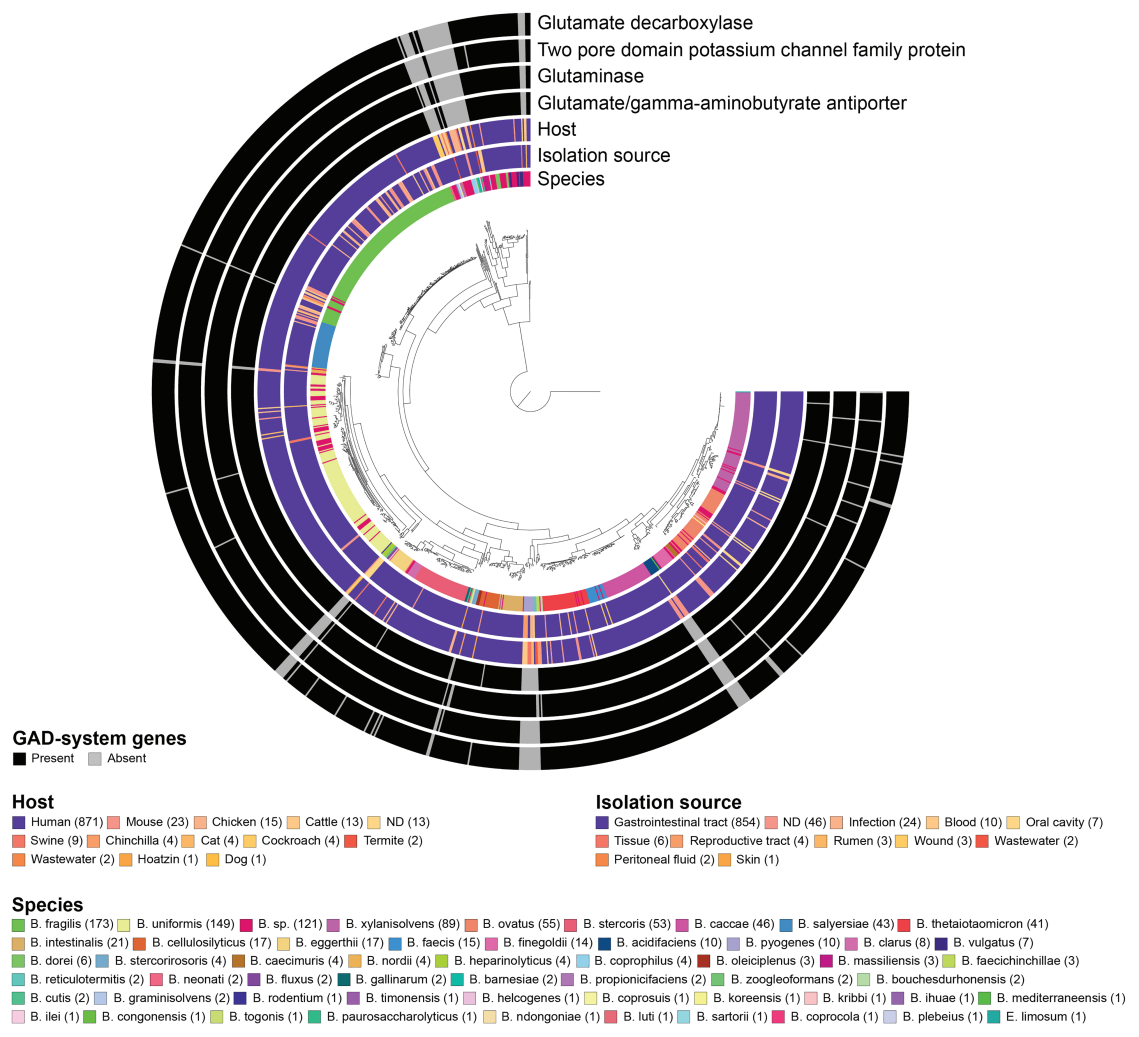
Prevalence of GAD-system genes in 961 *Bacteroides* genomes and association with species, host, and source of isolation. The phylogenomic tree was generated using 90 single-copy genes (SCG) specific to *Bacteroidetes*, and metadata were visualized *via* GToTree. *Eubacterium limosum* DSM 20543 genome was used to root the tree. All data are available in Supplementary Data File 3.

We next examined the prevalence of the GAD-system using a broad set of 961 *Bacteroides* genomes (~90% human isolates), representing more than 53 different species (most strains belonged to *B. fragilis* (18.0%), *B. uniformis* (15.5%), undefined species (“sp.”; 12.6%) and *B. xylanisolvens* (9.3%; Supplementary Data File 3). Overall, *gadB*, *gslA*, *gadC*, and the K^+^ channel-encoding gene are highly prevalent in *Bacteroides* and present in 92, 94, 93, and 92% of all genomes, respectively. About 90% of the strains possess all four genes, and among the remaining 10%, half of the genomes lack all four GAD-system genes entirely, thereby underlining the potential co-adaptation and functional relationship between these genes (Figure 3). Strains lacking the GAD-system genes belonged to *Bacteroides acidifaciens*, *Bacteroides barnesiae*, *Bacteroides coprophilus*, *Bacteroides graminisolvens*, *Bacteroides heparinolyticus*, *Bacteroides pyogenes*, *Bacteroides reticulotermitis*, and *Bacteroides zoogleformans* species (inclusion criteria: at least two genomes per species available; *gadB* prevalence ≤ 20%), most of which were initially isolated from animals or ecological niches other than the GIT (Figure 3; Supplementary Data File 3). A large majority of human isolates harbored *gadB* (96%; 870 strains), in contrast to mouse (48%, 23 strains), chicken (53%, 15 strains), cattle (38%, 13 strains), and swine (33%, 9 strains) isolates. None of the seven strains isolated from the oral cavity harbored *gadB* (Supplementary Data File 3). Interestingly, other genes involved in amino acid-dependent acid stress tolerance systems, including ornithine-, lysine-, tyrosine-, histidine-decarboxylases, and arginine deiminase (Guan and Liu, 2020) were absent in all *Bacteroides* genomes. Biodegradative arginine decarboxylase was only detected in two strains (Supplementary Data File 3). These data indicate that the GAD-system might constitute the only amino acid-dependent acid stress tolerance mechanism in *Bacteroides*.

### GABA Production Kinetics in *B. thetaiotaomicron* DSM 2079

**Figure 4:**
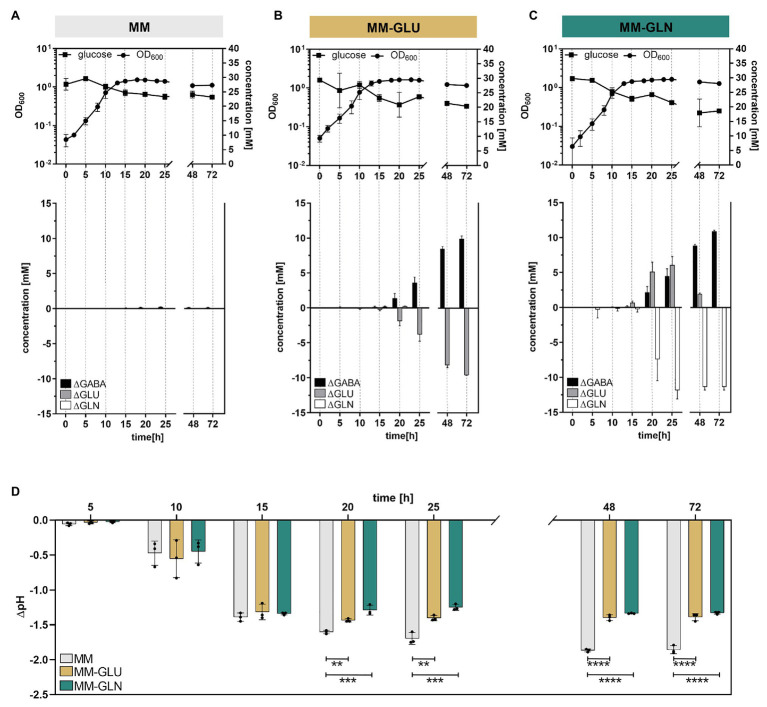
GABA production kinetic in the presence or absence of glutamine and glutamate in minimal medium (MM). Growth curves (log-scale) of *Bacteroides thetaiotaomicron* DSM 2079 cultivated in **(A)** MM, **(B)** MM-Glu, and **(C)** MM-Gln (*n* = 3). Changes in glucose, GABA, glutamine, and glutamate concentrations for 72 h are displayed. **(D)** Change in pH associated with *B. thetaiotaomicron* DSM 2079 growth for 72 h in MM, MM-Glu, and MM-Gln. Dots represent replicates; bars represent SD. Significances were calculated by one-way ANOVA test, including Tukey’s test: ^**^p < 0.01; ^***^p < 0.001; ^****^p < 0.0001. Data on metabolic acid production and absolute pH are available in Supplementary Figure S6.

Our *in silico* analysis revealed the presence of a yet unknown but highly conserved *gslA*/*gadB* operon in *Bacteroides* (and related taxa like *Parabacteroides*). This raised the question whether glutamine, the most abundant free amino acid in the human body and a major substrate of intestinal cells (Kim and Kim, 2017), could serve as a precursor for the production of GABA in *Bacteroides*. We performed growth kinetic tests over 72 h, by monitoring the growth of the model organism *B. thetaiotaomicron* DSM 2079 in MM supplemented with glutamine (11.86 ± 0.99 mM; MM-Gln) or glutamate (9.95 ± 0.30 mM; MM-Glu). Glutamine was confirmed as a secondary precursor of GABA, as its addition in the medium led to the production of glutamate, an intermediate step for the generation of GABA (Figure 4C). After 72 h, GABA was detected at a final concentration of 10.88 ± 0.15 mM in MM-Gln (Y_GABA/Gln_ = 1.03 ± 0.03; mol/mol), 9.95 ± 0.30 mM in MM-Glu (Y_GABA/Glu_ = 0.96 ± 0.03; mol/mol), and 0.16 ± 0.02 mM in MM alone (Figures 4A–C). The low production of GABA in the absence of added precursors was unexpected, and could have arisen from the conversion of α-ketoglutarate (a product of the citric acid cycle) to glutamate *via* the glutamate dehydrogenase (Glass and Hylemon, 1980). However, MM supplementation with 5 mM α-ketoglutarate failed to increase GABA levels under the tested conditions (data not shown).

Production of extracellular GABA started at the end of exponential growth phase (μM range at *t* = 15 h) and rapidly increased over time (mM range from *t* = 20 h onward), suggesting a non-growth associated production (Figures 4A–C). These data were investigated at the transcript level using the transcriptome database “Theta-Base,” showing a substantial increase of the expression of all four GAD-system genes during the stationary growth phase of *B. thetaiotaomicron* DSM 2079 (Supplementary Figure S4B). Addition of GABA precursors did not significantly affect the maximum growth rate, maximum cell density (Supplementary Table S2), or the entry to the stationary phase (not caused by substrate limitation; Figures 4A–C). However, carbohydrate metabolism was significantly affected by the presence of GABA precursors, with higher glucose consumption (Figures 4A–C) and organic acids production (i.e., formate, acetate, lactate, succinate, and propionate; Supplementary Figure S6) observed after 72 h in MM-Gln and MM-Glu compared to MM alone (*p* ≤ 0.0002 for all). Strikingly, the increased organic acid production observed in MM-Gln and MM-Glu did not result in an extensive pH drop (Supplementary Figure S6), on the contrary, acidification was significantly higher in MM from 20 h of growth onward (*p* < 0.01 for all; Figure 4D). A strong buffering effect was observed at pH ~4.9 in MM-Gln and MM-Glu, which coincided with the initiation of GABA production by *B. thetaiotaomicron* DSM 2079 (Supplementary Figure S6).

### Role of GABA Production Against Acid Stress in *B. thetaiotaomicron* DSM 2079

**Figure 5:**
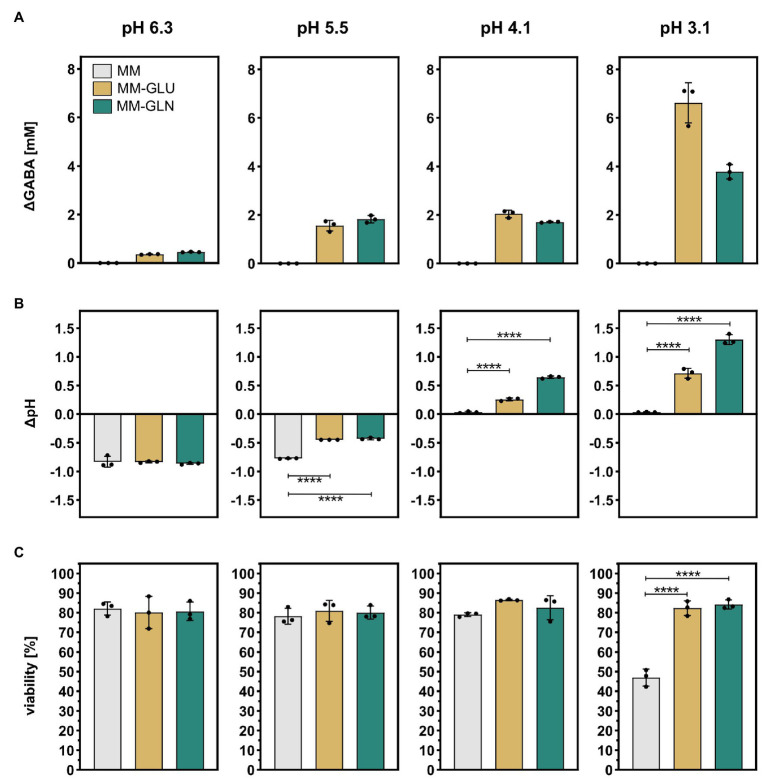
Effect of acid stress on GABA production, pH modulation, and cell viability in *B. thetaiotaomicron* DSM 2079. *B. thetaiotaomicron* DSM 2079 cells were incubated in MM, MM-Glu, or MM-Gln at pH 6.3, 5.5, 4.1, or 3.1 for 1 h (*n* = 3). **(A)** Concentration of GABA, **(B)** change in pH, and **(C)** viability are displayed. Dots represent replicates; bars represent SD. Significances were calculated by one-way ANOVA test, including Tukey’s test: ^****^p < 0.0001. Data on glucose consumption and metabolic acid production are available in Supplementary Table S3.

To confirm that GABA production increases *Bacteroides* cells viability under acidic conditions, *B. thetaiotaomicron* DSM 2079 was grown in MM until late exponential growth phase, harvested by centrifugation, and subsequently resuspended in MM-Gln, MM-Glu, or MM at four different pH each, ranging from 6.3 to 3.1, and incubated at 37°C for 1 h. In MM, GABA was not detected under any of the tested pH conditions. In the presence of GABA precursors, low GABA levels were produced in MM-Gln (0.45 ± 0.01 mM) and MM-Glu (0.36 ± 0.01 mM) at pH 6.3, whereas GABA production was maximal at the lowest pH of 3.1 (3.78 ± 0.25 mM in MM-Gln; 6.62 ± 0.68 mM in MM-Glu; Figure 5A). As expected from previous assays, GABA production concurred with an increase of the culture pH (ΔpH), with significant differences in ΔpH observed at pH 5.5 and below in MM-Gln vs. MM and MM-Glu vs. MM (*p* < 0.0001 for all; Figure 5B). The acidification of the cultures (negative ΔpH) observed at pH 5.5 and 6.3 concurs with glucose consumption and organic acids production (Supplementary Table S3). Finally, a significantly higher viability was measured at pH 3.1 in MM-Gln (84.1 ± 1.9%) and MM-Glu (82.3 ± 3.0%) compared to MM alone (47.0 ± 3.5%; *p* < 0.0001 for all; Figure 5C). Although not significant, at pH 4.1, a similar trend toward higher viability in MM-Gln (82.5 ± 5.0%) and MM-Glu (86.5 ± 0.3%) compared to MM (79.0 ± 0.8%) was observed. No significant differences between viabilities were detected at pH 5.5 and above (Figure 5C). Taken together, these data confirmed the crucial role of GABA-production as a mechanism of tolerance to highly acidic conditions in *Bacteroides*.

## Discussion

The neuroactive potential of metabolites produced by gut microbes has recently gained traction for their possible contribution to health and diseases, and as such, bacterially-derived GABA could represent one key neuro-immune modulator linking gut microbiota and mental health (Zheng et al., 2016; Strandwitz, 2018; Strandwitz et al., 2019; Valles-Colomer et al., 2019). Although the presence of *gadB* was previously reported in intestinal *Bacteroides*, a comprehensive analysis of the phenotypic, genotypic, and functional characteristics of GABA production was still lacking. This study presents the first in-depth screening of human intestinal *Bacteroides* to produce GABA, validating previously reported genetic predisposition *in vitro*, and demonstrating the high prevalence of GABA production, particularly among human gut isolates. Most strains tested (16 out of 17) produced GABA at concentrations ranging from 0.09 to 60.84 mM, comparable to levels observed in high GABA-producing *Lactobacillus* and *Bifidobacterium* strains (Barrett et al., 2012). We showed that levels of GABA produced were strain-specific, dependent on precursor availability and concentration, and stimulated by acidic conditions. Glutamate and glutamine were identified as primary and secondary precursors, respectively.

Importantly, we confirmed the beneficial role of GABA production as a mechanism of acid stress tolerance in *Bacteroides*, previously only reported for other bacterial taxa. Using the model organism *B. thetaiotaomicron* DSM 2079, we showed that production of extracellular GABA in minimal medium was not coupled to bacterial growth, but was induced during the late exponential growth phase as a response to medium acidification upon organic acid production. However, our data do not permit to (i) discard the possibility of intracellular GABA accumulation earlier during the growth phase (Karatzas et al., 2010) or (ii) fully discriminate between pH and growth phase as regulating factors of GABA production. In *E. coli*, both factors were shown to contribute to *gadA* and *gadB* regulation (Castanie-Cornet and Foster, 2001). Finally, GABA production by *B. thetaiotaomicron* DSM 2079 prevented medium acidification in batch cultures as compared to the GABA-free control, leading to a higher overall metabolism (i.e., higher glucose consumption and organic acids production), in part owing to a reduction of acid-induced cellular damage.

Analysis of the synteny and sequence similarities of the GAD-system genes revealed a high degree of conservation among *Bacteroides*. The GAD-system consists of a GAD (*gadB* ortholog; IPR010107), a glutaminase (*glsA* ortholog; IPR015868), a glutamate/GABA antiporter (*gadC* ortholog, IPR022520), and a K^+^ channel (IPR028325). The combination of *gadB* and *glsA* within the same operon was not previously described in *Bacteroides*, and constitutes an efficient intracellular proton-consuming system, scavenging two protons in total, one *via* free ammonia (*via* glutaminase) and another during GABA synthesis (*via* GAD; Lu et al., 2013; Pennacchietti et al., 2018). Our data confirmed that glutamine supplementation was at least as efficient as glutamate to protect *B. thetaiotaomicron* DSM 2709 cells upon acid stress exposure. K^+^ channels are the most diverse group of the ion channel family, involved in the creation and maintenance of a membrane potential in bacteria (Kuo et al., 2005), and contributing to essential physiological responses, i.e., antibiotic resistance, cell division, motility, environmental sensing, cell-cell communication, membrane transport, osmoregulation, and pH homeostasis (Padan et al., 1981; Wood, 2011; Benarroch and Asally, 2020). In *E.coli*, K^+^ influx was shown to favor GABA efflux by increasing the glutamate/GABA antiporter efficiency (Ma et al., 2012), further suggesting a functional relationship between the K^+^ channel-encoding gene and *gadC* in *Bacteroides*. Influx of K^+^ and glutamate (serving as a K^+^ counter ion) may also be driven by osmoregulation, as their intracellular accumulation is known to protect cells against osmotic stress (Wood, 2011).

Based on the conserved synteny, phylogeny, and the involvement of all GAD-system genes in pH homeostasis mechanisms, it is conceivable that the entire gene set could be co-regulated; a notion illustrated by similar transcription pattern of all GAD-system genes throughout growth as retrieved from “Theta-Base” (Ryan et al., 2020), but also by another recent study with *B. thetaiotaomicron* DSM 2079 where *gadB*, *glsA*, *gadC* and the K^+^ channel encoding gene were all downregulated when co-cultivated with *Phascolarctobacterium faecium* (Ikeyama et al., 2020).

Among the 961 *Bacteroides* strains analyzed, all four genes of the GAD-system were conjointly present in 90% of all genomes, or 96% when considering human gut isolates only. Since no other amino acid-dependent acid tolerance mechanisms were present in all genomes, the high prevalence of the GAD-system denotes an essential function for *Bacteroides*, especially in human gut isolates. Indeed, most strains lacking the GAD-system were obtained from animals (i.e., mouse, chicken, cattle, and swine, among others), corroborating another study where the absence of *gadB* and *gadC* could help discriminating chicken *Bacteroides* strains from human gut isolates (Kollarcikova et al., 2020). This evidence suggests host-specific adaptation of *Bacteroides* under distinct ecological constrains (e.g., diet, host physiology, and natural microbial community), potentially resulting in a loss-of-function (Hottes et al., 2013) of the GAD-system in animal isolates. The human proximal colon, where most fermentation occurs, possesses a lower pH (~5.7) than the cecum of chicken (~6.5) and mice (~6.5), the proximal colon of pigs (~6–6.5), or the rumen and colon of cattle (~6–6.5; Fallingborg, 1999; Merchant et al., 2011; Mao et al., 2015; Poeker et al., 2019; Herwig et al., 2020). These differences in environmental pH among host intestinal tracts could explain, at least in part, the essential role and prevalence of the GAD-system in *Bacteroides* human isolates.

The GAD-system may provide advantage to *Bacteroides* spp. to cope with the dynamic physicochemical conditions of the human large intestine. On one hand as a potential contributor to osmoregulation to survive osmotic shifts resulting from water absorption and transport of ions by colonic epithelial cells (Costongs et al., 1985; Cesar et al., 2020); on the other hand to cope with the dynamic pH across the large intestine. On average, the pH of the human adult colon ranges from 5.7 (proximal) to 6.7 (distal; Fallingborg, 1999), but varies among individuals with values as low as 5.0 reported in healthy subjects (Koziolek et al., 2015). The colonic pH can also fluctuate in response to diet- and host-associated factors, e.g., *via* food consumption and microbial fermentation of dietary fibers (Naaeder et al., 1998; Chung et al., 2007), inflamed colonic mucosa (Nugent et al., 2001), or micro acidic intestinal environments (Vroom et al., 1999; Boleij and Tjalsma, 2012). Throughout the human lifespan, *Bacteroides* spp. might also benefit from the GAD-system, especially early in life when dominant lactate-producing taxa such as *Bifidobacterium* lower the intestinal pH through their metabolic activity (Henrick et al., 2018). *Bacteroides* are early colonizers of the infant gut (Palmer et al., 2007), but have been considered as acid-sensitive due to their poor growth under mildly acidic conditions *in vitro* (i.e., pH 5.5; Duncan et al., 2009). Our data indicate that GABA production successfully mitigates acid-induced cellular damage, eventually allowing *Bacteroides* to proliferate in the GIT once pH conditions are favorable.

*Bacteroides*, one of the most abundant and prevalent genera of the GIT (Pokusaeva et al., 2017), are well-adapted to the conditions in the human gut, with remarkable metabolic potential and nutritional flexibility to consume both diet- and host-derived carbohydrates (e.g., human milk oligosaccharides, starch, xylan, pectin, and mucins; Salyers et al., 1977; Marcobal and Sonnenburg, 2012), thereby allowing a stable and resilient colonization of this highly dynamic ecosystem throughout the host’s lifespan (Human Microbiome Project Consortium, 2012). In turn, their metabolic activity contributes to the production and/or transformation of a wide range of bioactive compounds (e.g., acetate, propionate, succinate, H_2_, and bile acids) that shape the symbiotic relationship between *Bacteroides*, the host, and other microbes (Chow et al., 2010; Yao et al., 2018). Here, we demonstrated that *Bacteroides* can also produce the neuroactive GABA, and thus may contribute to the regulation of the GABAergic system in humans. This complex system is in the process of being understood: while produced GABA may serve as additional carbon source for some gut commensal bacteria (Strandwitz et al., 2019) and act as signaling molecule between bacteria (Dagorn et al., 2013), it may also interact with the vast amounts of GABA receptors present in the GIT (Seifi and Swinny, 2019; Zhao et al., 2020) and stimulate the immune system (Zhao et al., 2020). This may explain the positive effects of GABA-enriched foods in attenuating anxiety and insomnia (Yu et al., 2020), and the correlation between bacterially-derived GABA and/or GAD-system genes in mental health, such as reported for autism (Kang et al., 2018; Averina et al., 2020) and depression (Zheng et al., 2016; Strandwitz et al., 2019; Valles-Colomer et al., 2019).

## Data Availability Statement

The original contributions presented in the study are included in the article/Supplementary Material, further inquiries can be directed to the corresponding author.

## Author Contributions

NO and BP designed the experiments. AK and BP implemented the UPLC method in the laboratory. CL, LB, and TW gave inputs on single strain culturing. NO, KY, DM, and LB performed the experiments. NO, FC, FAC, CL, and BP performed the data analysis. CB and CL provided financial support. NO and BP wrote the manuscript. All authors critically reviewed the manuscript. All authors contributed to the article and approved the submitted version.

### Conflict of Interest

Authors LB and TW were employed by the company PharmaBiome AG.

The remaining authors declare that the research was conducted in the absence of any commercial or financial relationships that could be construed as a potential conflict of interest.
