# Supplementary material for: GABA Production by Human Intestinal Bacteroides spp.: Prevalence, Regulation, and Role in Acid Stress Tolerance
Source: Front Microbiol. 2021 Apr 15;12:656895. doi: 10.3389/fmicb.2021.656895 (PMC8082179; doi:10.3389/fmicb.2021.656895)
Supplement: Supplementary file 1 [file Data_Sheet_1.zip › Supplementary material/Supplementary Figures and Tables.docx]

**Supplementary data:**

**Table S1: Change in GABA (Δ GABA) and glutamate (Δ glutamate) concentrations in *Bacteroides* cultures grown in mYCFA and mYCFA-Glu for 48 h.**

| **Species** | **Strain** | **Medium** | **Δ GABA (mM)** | | **Δ glutamate (mM)** | |
| --- | --- | --- | --- | --- | --- | --- |
|  |  |  | **Mean** | **SD** | **Mean** | **SD** |
| *B. caccae* | DSM 19024 | mYCFA | 0.85 | 0.08 | -1.09 | 0.15 |
|  |  | mYCFA-Glu | 0.78 | 0.05 | -2.51 | 2.24 |
| *B. dorei* | PB-SNPAX | mYCFA | 0.74 | 0.02 | -1.15 | 0.41 |
|  |  | mYCFA-Glu | 8.80 | 0.94 | -18.91 | 1.80 |
| *B. faecis* | DSM 24798 | mYCFA | 6.91 | 0.57 | -7.70 | 0.10 |
|  |  | mYCFA-Glu | 12.29 | 2.22 | -14.47 | 1.06 |
| *B. faecis* | PB-SESWS | mYCFA | 6.56 | 0.69 | -7.39 | 0.11 |
|  |  | mYCFA-Glu | 46.59 | 1.81 | -58.51 | 0.75 |
| *B. fragilis* | DSM 2151 | mYCFA | 0.09 | 0.01 | 0.02 | 0.00 |
|  |  | mYCFA-Glu | 0.12 | 0.01 | -4.73 | 3.02 |
| *B. fragilis* | PB-SZSJC | mYCFA | 6.38 | 0.74 | -7.39 | 0.12 |
|  |  | mYCFA-Glu | 37.48 | 2.90 | -48.89 | 1.30 |
| *B. intestinalis* | DSM 17393 | mYCFA | 2.06 | 0.48 | -2.00 | 0.41 |
|  |  | mYCFA-Glu | 17.95 | 0.37 | -22.72 | 2.16 |
| *B. ovatus* | DSM 1896 | mYCFA | 7.68 | 0.14 | -7.73 | 0.02 |
|  |  | mYCFA-Glu | 54.07 | 4.20 | -64.20 | 0.79 |
| *B. plebeius* | PB-SLKZP | mYCFA | n.d. | n.d. | 0.93 | 0.42 |
|  |  | mYCFA-Glu | n.d. | n.d. | 4.68 | 1.77 |
| *B. thetaiotaomicron* | DSM 2079 | mYCFA | 6.35 | 0.19 | -6.85 | 0.17 |
|  |  | mYCFA-Glu | 16.39 | 0.86 | -22.38 | 1.02 |
| *B. uniformis* | PB-SWTWH | mYCFA | 1.68 | 0.04 | -1.59 | 0.02 |
|  |  | mYCFA-Glu | 0.66 | 0.03 | 1.31 | 1.42 |
| *B. uniformis* | PB-SARUR | mYCFA | 2.08 | 0.01 | -1.61 | 0.27 |
|  |  | mYCFA-Glu | 4.21 | 0.52 | -8.91 | 2.60 |
| *B. uniformis* | PB-SMSXL | mYCFA | 2.81 | 0.08 | -2.30 | 0.06 |
|  |  | mYCFA-Glu | 3.09 | 0.57 | -9.41 | 5.21 |
| *B. uniformis* | DSM 6597 | mYCFA | 3.70 | 0.86 | -3.84 | 0.71 |
|  |  | mYCFA-Glu | 11.68 | 2.60 | -16.85 | 2.86 |
| *B. vulgatus* | DSM 1447 | mYCFA | 0.28 | 0.10 | -0.39 | 0.18 |
|  |  | mYCFA-Glu | 0.35 | 0.14 | -2.59 | 0.84 |
| *B. vulgatus* | PB-SZEJJ | mYCFA | 1.01 | 0.35 | -1.67 | 1.09 |
|  |  | mYCFA-Glu | 3.20 | 0.88 | -9.40 | 3.37 |
| *B. xylanisolvens* | DSM 18836 | mYCFA | 7.35 | 0.29 | -7.75 | 0.06 |
|  |  | mYCFA-Glu | 60.84 | 0.85 | -68.08 | 0.09 |

n.d.: not detected

n.a.: not applicable

mYCFA-Glu: mYCFA supplemented with 61 mM glutamate

**Table S2: Effect of glutamate (MM-Glu) and glutamine (MM-Gln) on the maximum specific growth rate (µ_max_) and cell density of *Bacteroides thetaiotaomicron* DSM 2079.** No significant difference (*p*>0.05) were observed for µ_max_ and maximum cell density among the different media compositions.

| **Medium** | **µ_max_ (h^−1^)** | | **max. cell density (OD_600_)** | |
| --- | --- | --- | --- | --- |
|  | **Mean** | **SD** | **Mean** | **SD** |
| MM | 0.31 | 0.02 | 1.50 | 0.02 |
| MM-Glu | 0.26 | 0.01 | 1.58 | 0.05 |
| MM-Gln | 0.32 | 0.05 | 1.60 | 0.07 |

MM: minimal medium

MM-Glu: minimal medium supplemented with 10 mM glutamate

MM-Gln: minimal medium supplemented with10 mM glutamine

**Table S3: Change in glucose (Δ glucose) and total organic acid (Δ organic acids) concentrations measured in supernatants of *Bacteroides thetaiotaomicron* DSM 2079 incubated in different media (MM, MM-Glu and MM-Gln) at different pH (6.3, 5.5, 4.1, 3.1) for 1 h.**

| **pH** | **Medium** | **Δ glucose (mM)** | | **Δ organic acids (mM)** | |
| --- | --- | --- | --- | --- | --- |
|  |  | **Mean** | **SD** | **Mean** | **SD** |
| 6.3 | MM | -4.00 | 0.55 | 4.78 | 0.39 |
|  | MM-Glu | -2.70 | 0.16 | 6.02 | 0.11 |
|  | MM-Gln | -3.80 | 0.14 | 5.72 | 0.07 |
| 5.5 | MM | -2.25 | 0.48 | 2.28 | 0.07 |
|  | MM-Glu | -1.78 | 0.25 | 3.21 | 0.36 |
|  | MM-Gln | -3.21 | 0.19 | 2.76 | 0.07 |
| 4.1 | MM | -0.89 | 0.34 | 0.42 | 0.20 |
|  | MM-Glu | -2.89 | 2.74 | 0.39 | 0.19 |
|  | MM-Gln | -2.41 | 0.45 | 0.96 | 0.03 |
| 3.1 | MM | -0.69 | 0.33 | n.d. | n.a |
|  | MM-Glu | -1.49 | 0.52 | n.d. | n.a. |
|  | MM-Gln | -1.59 | 0.34 | 0.30 | 0.09 |

MM: minimal medium

MM-Glu: minimal medium supplemented with 10 mM glutamate

MM-Gln: minimal medium supplemented with10 mM glutamine

n.a.: not applicable

n.d.: not detected

**
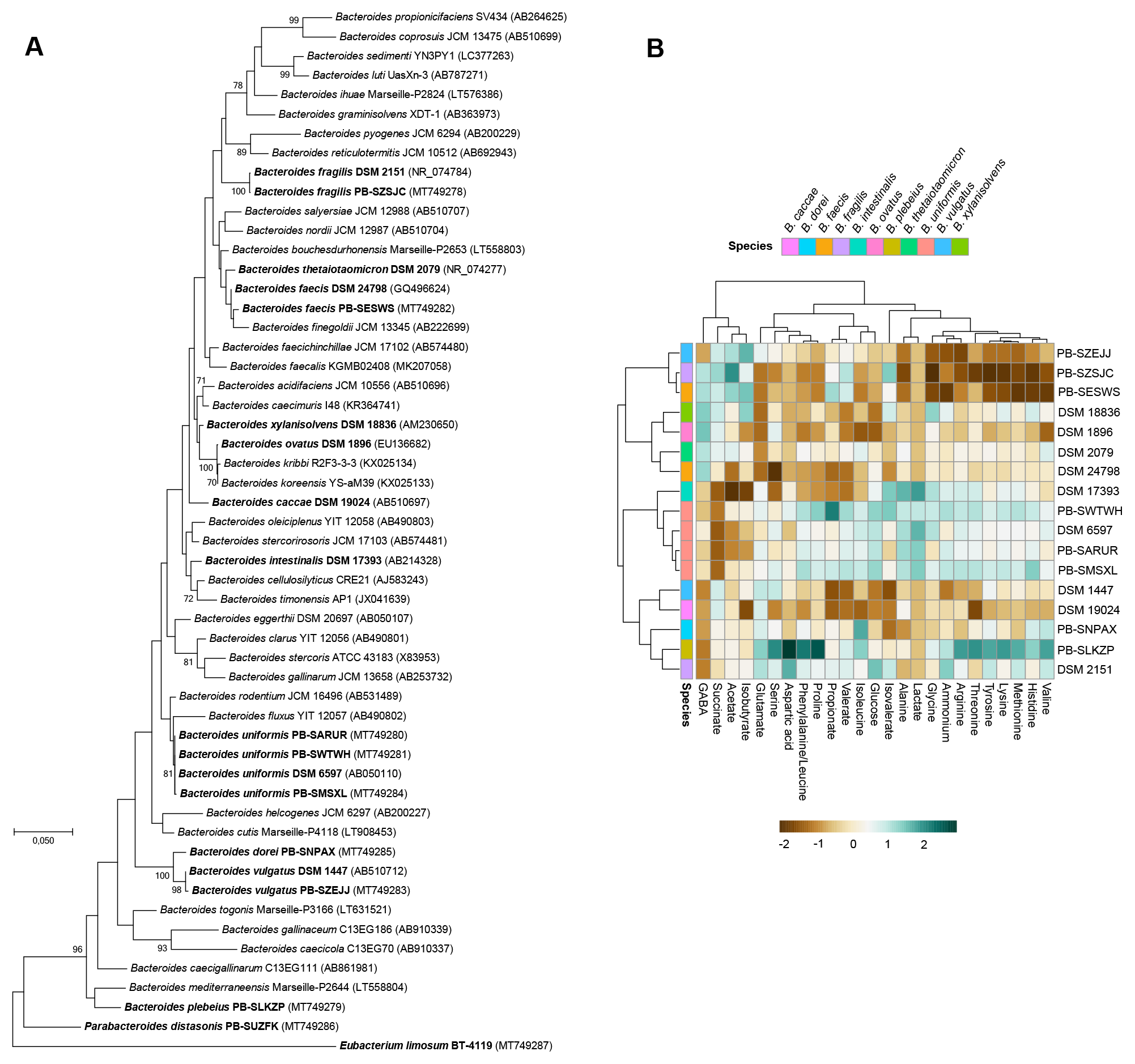
**

**Figure S1: Genetic and metabolic diversity of *Bacteroides* strains screened for GABA production *in vitro.*** (A) 16S rRNA gene sequence-based phylogenetic tree of *Bacteroides* strains and other tested strict anaerobes. Strains tested *in vitro* are indicated in bold. The tree was constructed with MEGA7 using the Maximum Likelihood method, and distances were computed using the Jukes-Cantor correction model (1000 bootstraps replicates). (B) Heatmap of substrates and bacterial metabolites in mYCFA after 48 h of cultivation. Data was scaled to z-scores and Pearson correlation was used to calculate rows and columns distance for hierarchical clustering. Absolute metabolite concentrations are available in **Supplementary Data File 1**

**
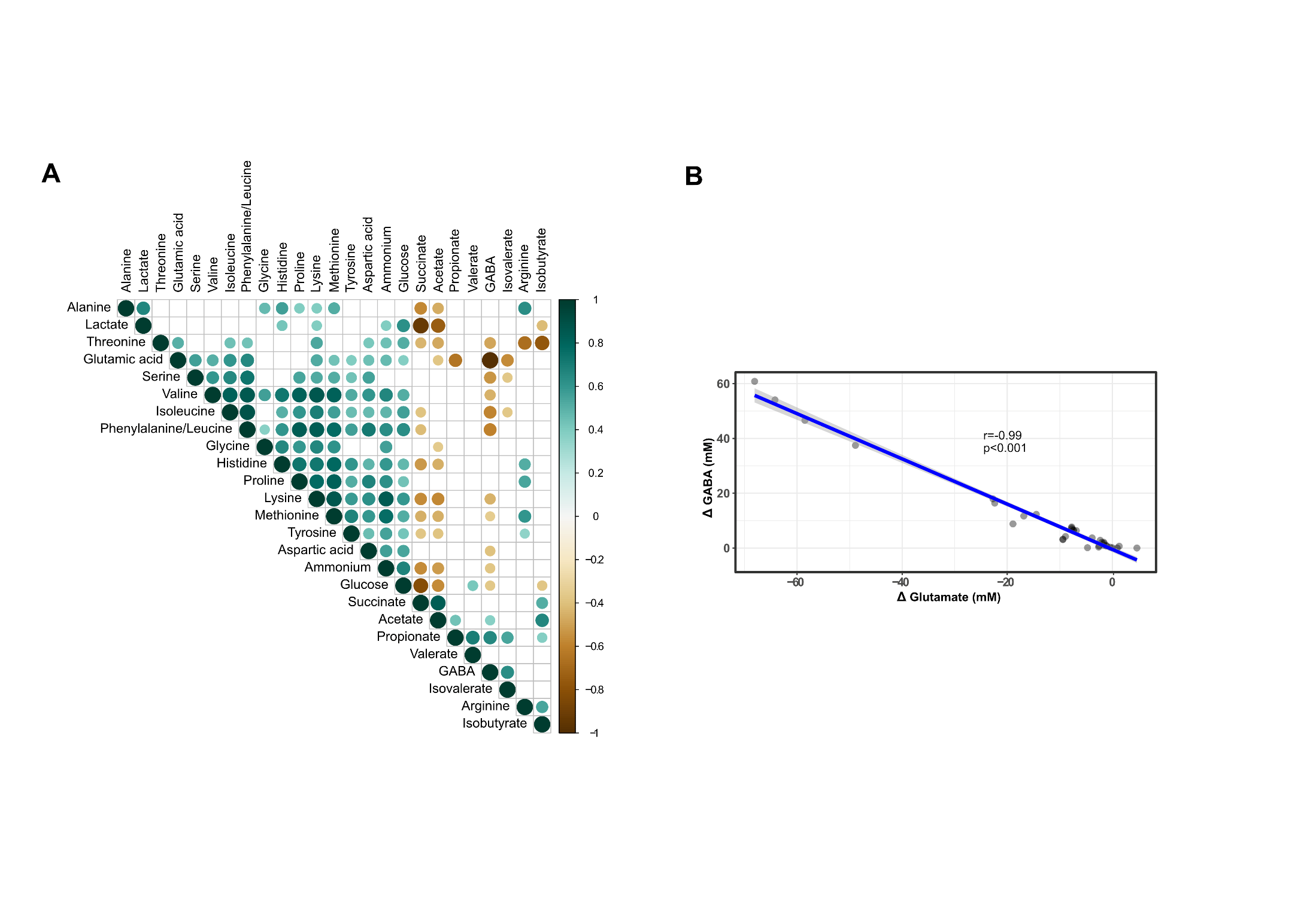
**

**Figure S2:** **Correlation between metabolites produced and substrates consumed during 48 h incubation of *Bacteroides* strains in mYCFA and mYCFA-Glu. (A)** Pearson correlation matrix of all metabolites with significant correlations indicated as dots (*p*<0.05). Positive *r* values (green) denote a positive correlation, and negative *r* values (brown) denote a negative correlation. Agmatine, asparagine, butyrate, cadaverine, glutamine, histamine, ornithine, putrescine, tryptamine, tryptophan and tyramine were not detected. **(B)** Pearson correlation between change in glutamate (Δ glutamate) and GABA (Δ GABA) concentration.

.
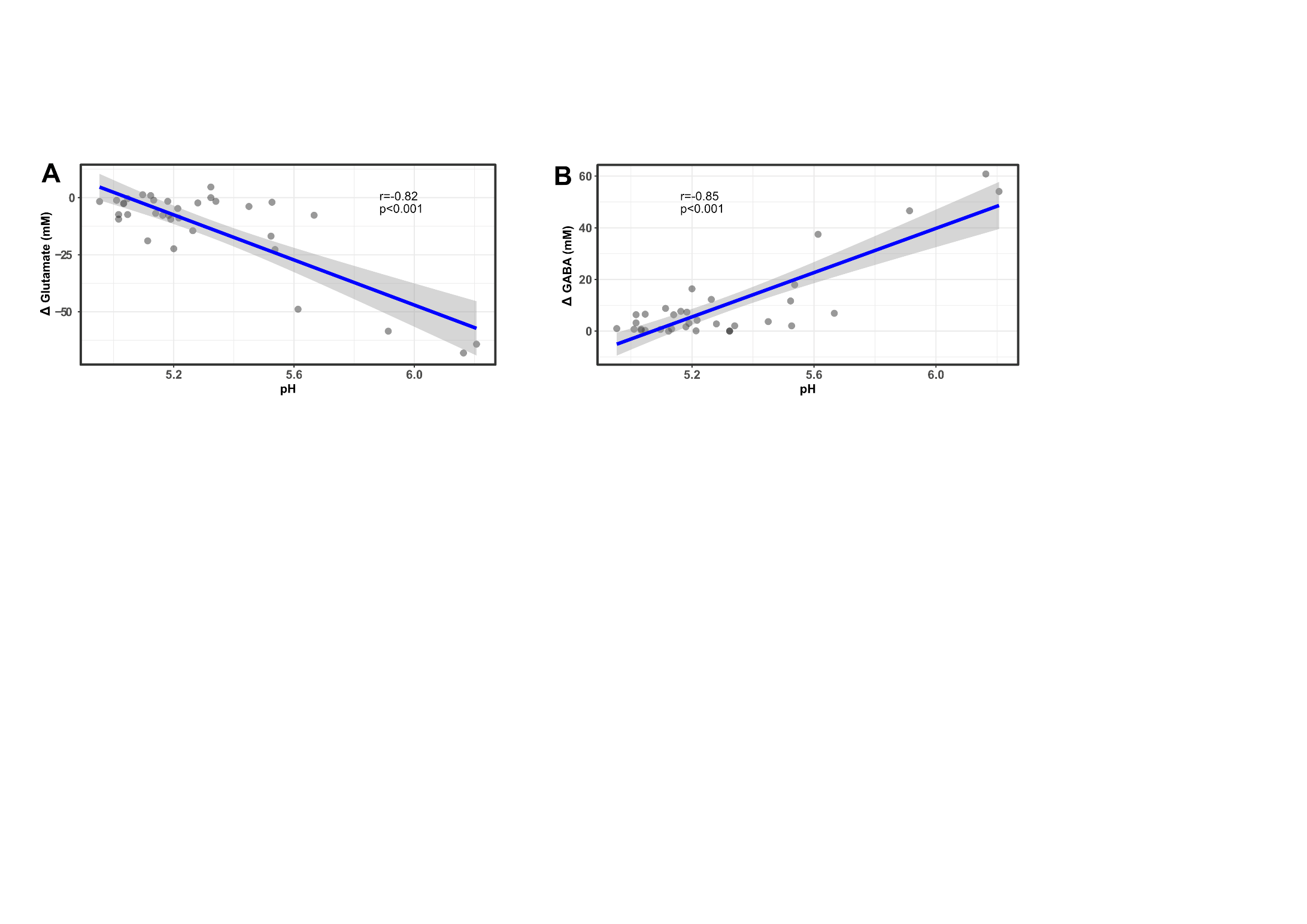


**Figure S3:** **Pearson** **correlation between (A) change in glutamate concentration (Δ glutamate) and pH, and (B) GABA production (Δ GABA) and pH during 48 h incubaction of *Bacteroides* strains grown in mYCFA and mYCFA-Glu.**


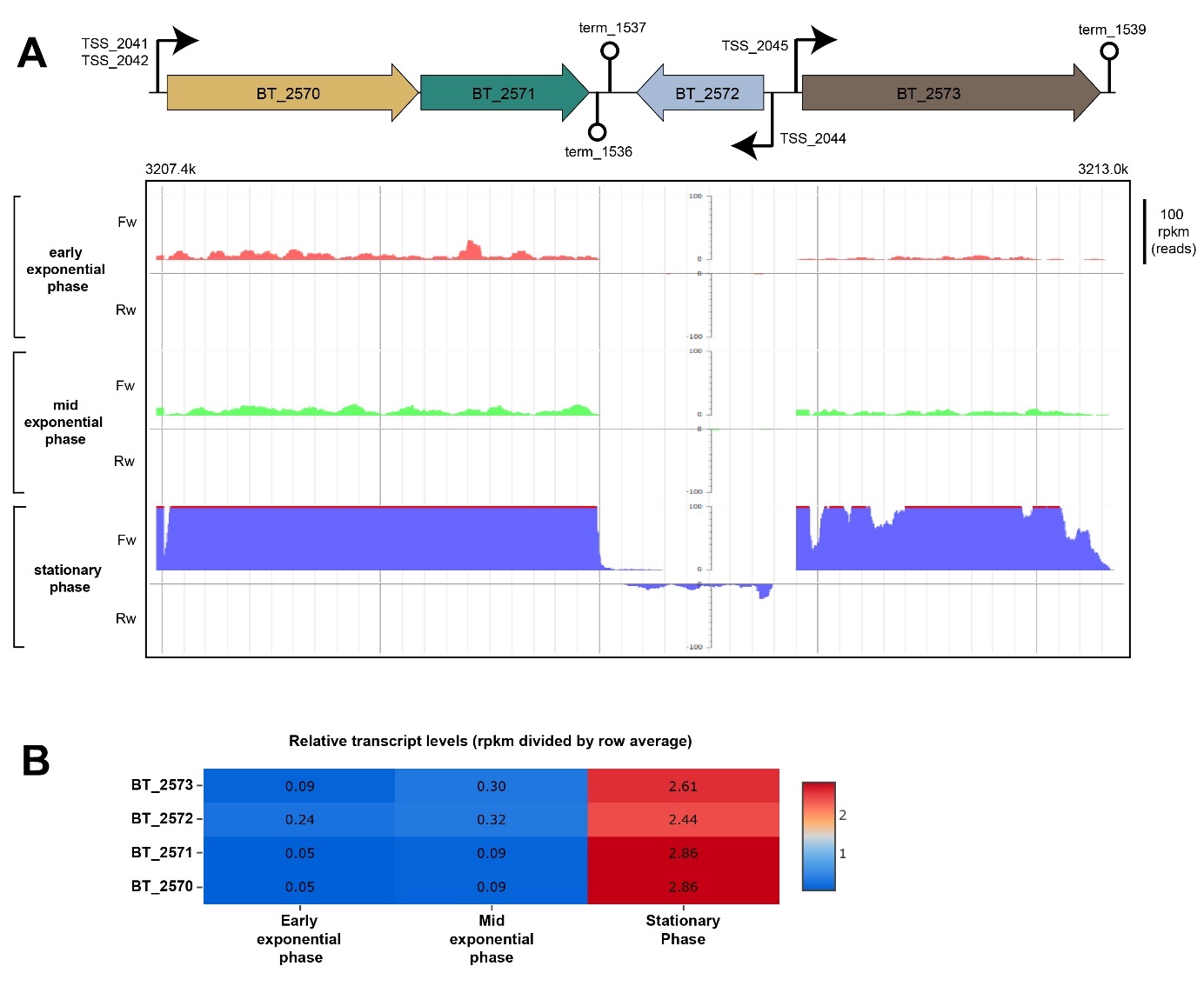


**Figure S4:** **Transcript features of the GAD-system genes in** ***B. thetaiotaomicron* DSM 2079 as determined via ‘Theta-Base’. (**A) Transcription start site (TSS) and transcription terminators (term) are indicated, confirming the presence of a *gadB/glsA* operon. Transcript levels of BT_2570 (glutamate decarboxylase), BT_2571 (glutaminase), BT_2572 (potassium channel), and BT_2573 (glutamate/GABA antiporter) in early exponential phase, mid-exponential phase and stationary phase is shown in the read coverage plots below (one representative replicate out of three is depicted). Detailed growth conditions and kinetics reported by Ryan and colleagues (2020). rpkm: average reads per kilobase of transcript per million mapped reads (max. 100 displayed). Fw: Leading strand. Rw: Lagging strand. (B) Relative transcript levels of the GAD-system genes at different growth phase (average of all three replicates). ‘Theta-Base’ is available at https://bacteroides.helmholtz-hzi.de/.


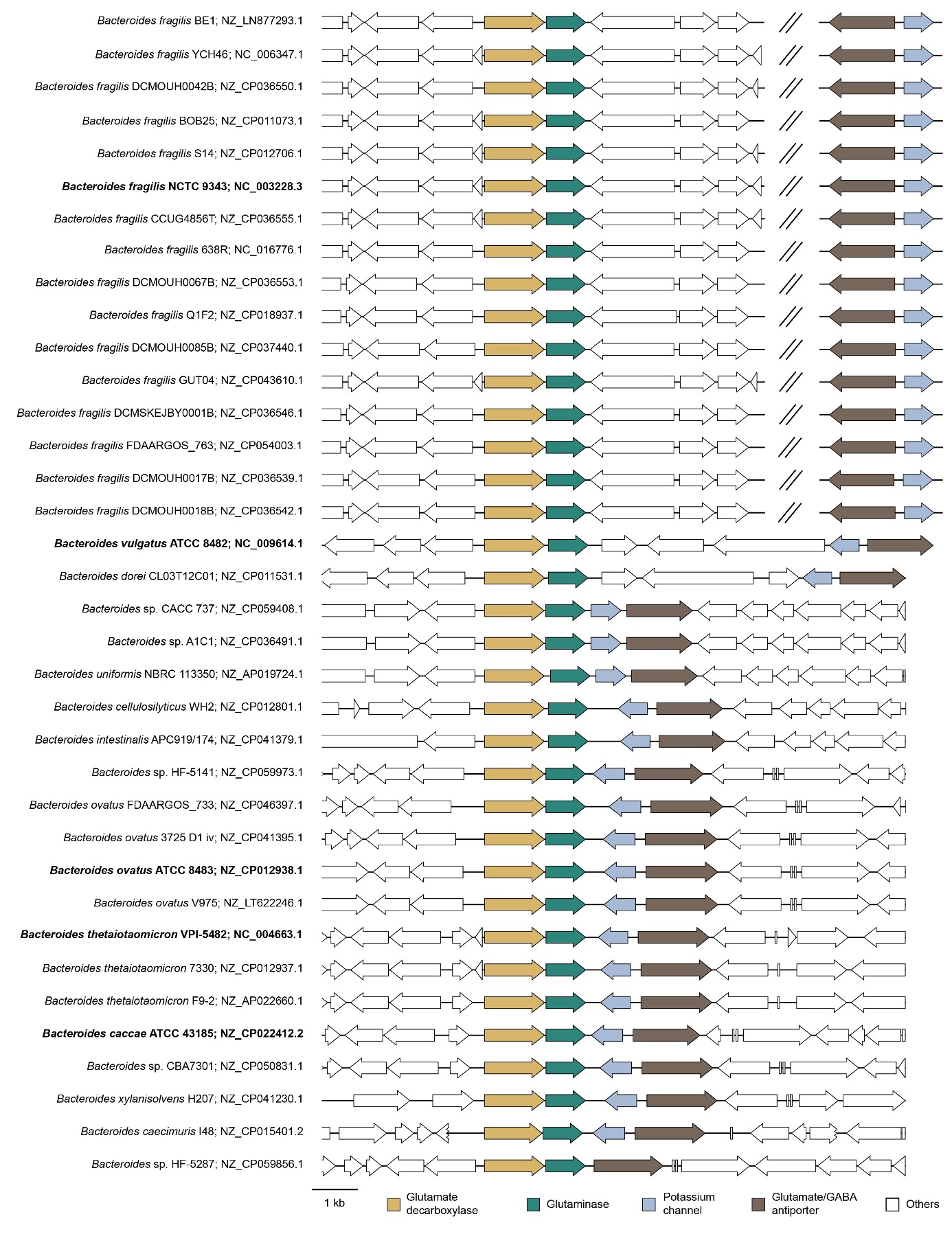


**Figure S5: Synteny** **of *gadB* and surrounding GAD-system genes in all *Bacteroides* strains with complete circular genome (n=40).** Four strains (*B. heparinolyticus* F0111, *B. zoogleoformans* ATCC 33285, *B. coprosuis* DSM 18011, *B. helcogenes* P 36-108) do not harbor *gadB* and are not displayed. The following genes are highlighted: glutamate decarboxylase, glutaminase, glutamate/GABA antiporter and potassium channel. Strains tested *in vitro* are indicated in bold.


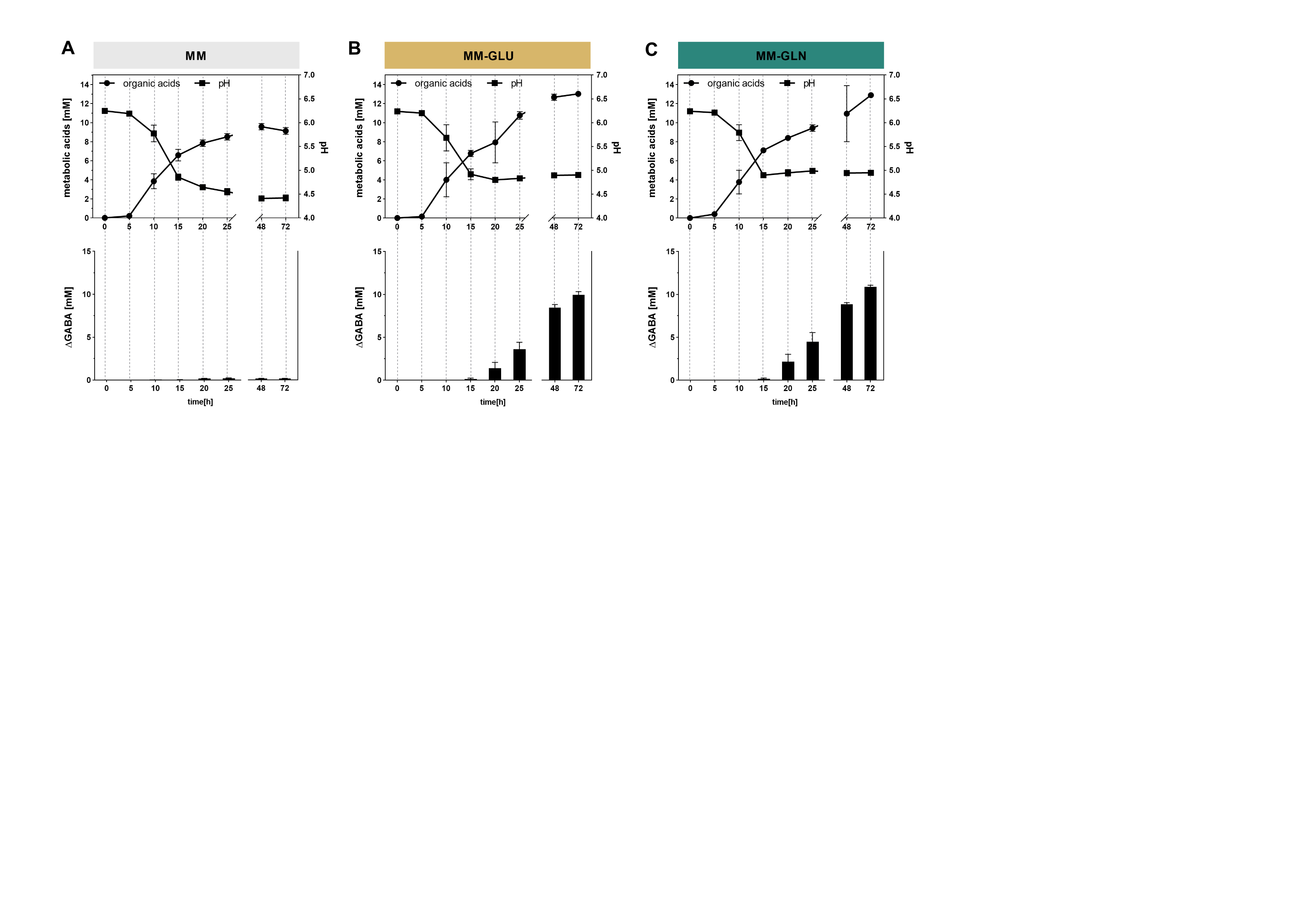
**Figure S6: GABA production kinetics and pH change in the presence of glutamine and glutamate.** *B. thetaiotaomicron* DSM 2079 was cultivated in (A) MM, (B) MM-Glu and (C) MM-Gln (n=3). Change of pH, total organic acids (*i.e.* formate, acetate, lactate, succinate and propionate), and GABA over a period of 72 h are displayed.
